# Plant Growth Regulators and Activated Charcoal Selectively Affect Phenylethanoid and Flavone Glycoside Accumulation in *Sideritis scardica* Griseb. Tissue Culture

**DOI:** 10.3390/plants12132541

**Published:** 2023-07-04

**Authors:** Kalina Danova, Jasmina Petreska Stanoeva, Ina Aneva, Kalina Alipieva, Marina Stefova

**Affiliations:** 1 Institute of Organic Chemistry with Centre of Phytochemistry, Bulgarian Academy of Sciences, Acad. Georgi Bonchev Str., bl.9, 1113 Sofia, Bulgaria; kalina.alipieva@orgchm.bas.bg; 2 Institute of Chemistry, Faculty of Natural Science and Mathematics, Ss. Cyril and Methodius University, Arhimedova 5, 1000 Skopje, North Macedonia; jasmina.petreska@pmf.ukim.mk (J.P.S.); marinaiv@pmf.ukim.mk (M.S.); 3 Institute of Biodiversity and Ecosystem Research, Bulgarian Academy of Sciences, 2 Gagarin Str., 1113 Sofia, Bulgaria; ina.aneva@abv.bg

**Keywords:** *Sideritis scardica* Griseb. in vitro culture, activated charcoal, plant growth regulators, flavone glycosides, phenylethanoids

## Abstract

*Sideritis scardica* Griseb. is a Balkan endemic species traditionally used for the treatment of pulmonary emphysema and angina pectoris. Recent research has also shown its phytotherapeutic potential as an anticancer and neuroprotective agent. These findings, as well as the endangered status of the species in its wild habitats, have motivated the present research on application of plant cell tissue and organ culture for the purposes of both valuable germplasm conservation and secondary metabolites production. Shoot cultures of the plant were initiated from sterile germinated seeds and the effects of activated charcoal (AC), as well benzyl adenine and 1-naphthaleneacetic acid treatments, were experimented. The phenolic profile analysis was performed by HPLC/DAD/MS^n^. Comparison with samples collected from wild plants in their natural habitat was performed. It was established that in vitro multiplication induced by plant growth regulators (PGRs) was accompanied by a higher impairment of leaf morphology and trichome formation, as well as by the occurrence of plantlet hyperhydricity and callus formation, as compared with the AC treatments. Shoot culture-derived plant material was shown to produce two phenylethanoids and five flavone glycosides, not detected in the wild collected plant material. In addition, the two types of in vitro culture treatments led to the stimulation of either flavone glycosides or phenylethanoids in the in vitro cultivated plants. Thus, AC stimulated, to a higher extent, flavone glycosides’ accumulation, leading to an elevated flavone/phenylethanoid ratio, as compared with PGR treatments.

## 1. Introduction

The *Sideritis* genus (Lamiaceae) consists of over 150 species of herbal and shrub habitus [1].

The first mention of the medicinal application of *Sideritis* species dates as far as the 1st century in the Dioscorides book “De Materia Medica” [2]. As mentioned in the ancient source, the plant was used to treat wounds made by metal weapons; hence, the name of the genus derives from the Greek “*sideros*”—meaning “iron”. In traditional medicinal practices, the decoction or the infusion of the aerial parts have been applied either internally or externally as anti-inflammatory, anti-ulcerative, antimicrobial, vulnerary, anti-spasmodic, anti-convulsant, analgesic, and carminative remedies [3]. A systematic study survey of 47 representatives of the *Empedoclea* section of genus *Sideritis* established records of antioxidant, antimicrobial and antibacterial, anti-inflammatory, antifungal, and cytotoxic activities in the different species [4]. In addition, other prospective phytopharmacological applications of *Sideritis* species were found, such as antifeedant [5,6] and anti-HIV activities [7,8] in *S. akmanii* (Z. Aytac, M. Ekici, and A. Donmez (Daǧçayı)). Activities, such as bifidogenic, cytotoxic, preventative of osteoporosis, protective effect on bone mineral density and strength, and beneficial activity in Alzheimer‘s disease were established for *S. euboea* Heldr. (Greek mountain tea) [4]. *S. scardica* Griseb., belonging to the *Empedoclea* section of the genus, is endemic to the Balkan peninsula. It has been designated as Greek mountain tea, Shepherd’s tea, Ironwort, Mursalski tea, Pirinski tea, or Caj Mali, depending on its area of distribution [9]. Traditional use of the herb includes the preparation of an infusion or decoction of the aerial parts of the plant, both individually or in combination with other medicinal plants for a wide array of ailments, such as asthma, bronchitis, lung emphysema, common cold, angina pectoris, rhinitis, for anemia prevention, chronic kidney disease, prostate inflammation, as antirheumatic amongst other applications [10]. Pharmacological studies on different preparations of the species have shown its potential in treating Alzheimer’s disease [9], as well as being an anticancer agent [11]. Moreover, studies have shown a lack of toxicity and a favorable mutagenic profile, confirming the safety of the plant, as evidenced in its wide traditional application [12].

The phytochemical constituents present in the plant determine the multitude of pharmacological activities in the different *Sideritis* species. In a review article, Fraga [1] presents a survey on the chemical composition of the Mediterranean representatives of the genus. The main secondary metabolites are mono- and sesquiterpenoid compounds (iridoid glycosides and eudesmane sesquiterpenes), diterpenes (labdane and isokaurene types), triterpenes, and sterols. Another main class of metabolites are phenolic compounds, such as flavones and their glycosides (7-*O*-glycosides of 8-hydroxyflavones, 7-*O*-glycosides of common flavones apigenin, luteolin, and chrysoeriol), coumarins, derivatives of caffeic, *p*-coumaric, chlorogenic, protocatechuic and quinic acids, as well as phenylethanoid glycosides, such as verbascoside, isoverbascoside, leucoseptoside A, martynoside, lavandulifolioside, forsythoside B, alyssonoside, and echinacoside.

The *Sideritis* species, distributed throughout the Balkan Peninsula, consists of representatives of the *Empedoclea* (*S. scardica*, *S. raeseri* and *S. syriaca*) and *Hesiodia* (*S. montana* and *S. lanata*) sections of the *Sideritis* sub-genus [13]. In a work by Stanoeva et al. [13], the polyphenolic profile of forty-two samples of Balkan representatives of the genus (*S. scardica*, *S. raeseri*, *S. syriaca*, *S. taurica,* and *S. lanata*) has been studied. The survey showed that eight of all the identified phenolic compounds were common for all samples (5-caffeoylquinic acid, lavandulifolioside, verbascoside, isoscutellarein 7-*O*-allosyl(1→2)glucoside, hypolaetin 7-*O*-[6′′′-*O*-acetyl]-allosyl(1→2)glucoside, isoscutellarein 7-*O*-[6′′′-*O*-acetyl]-allosyl(1→2) glucoside, 3′-*O*-methylhypolaetin 7-*O*-[6′′′-*O*-acetyl]-allosyl(1→2)glucoside, and 4′-*O*-methylhypolaetin 7-*O*-[6′′′-*O*-acetyl]-allosyl-(1→2)-[6″-*O*-acetyl]-glucoside), representing 50–80% of the total phenolic content of *S. scardica*, *S. raeseri*, *S. syriaca,* and *S. taurica* and up to 90% of the one of *S. lanata.*

Previous tissue culture experiments on *S. scardica* have evaluated the effect of plant growth regulators on in vitro propagation and ex vitro adaptation of the plant [14]. In our preliminary screening studies on the effect of different types of treatments (including plant growth regulators and activated charcoal), on the developmental patterns, total phenolics productivity, and photosynthetic activity of *S. scardica* shoot cultures, promising results on the effect of activated charcoal were established [15]. The effect of plant growth regulators on the total phenolic and flavonoids content, hydrogen peroxide levels, low molecular antioxidants (ascorbate and glutathione), as well as antioxidant enzymes, have also been studied for this species in tissue culture conditions in our previous research [16]. Studies on other representatives of the genus in vitro were performed by Sarropoulou and Maloupa [17], who investigated the effect of activated charcoal, salicylic acid, and α- and β-cyclodextrins on the in vitro development of *S. raeseri* (Boiss et Heldr). In further research on the in vitro culture of *S. raeseri* subsp. *attica*, Bertsouklis et al. [18] studied the effect of plant growth regulators on the in vitro multiplication and subsequent ex vitro adaptation of the plant. Micropropagation of *S. pisidica* (Boiss et Heldr). Apud Bentham has been performed, reporting on the effect of plant growth regulators on this species [19].

The aim of the present study was to characterize the individual compounds with phenolic structure, produced by *S. scardica* in shoot culture conditions with special attention on distinguishing between the principally different tissue culture treatments, i.e., plant growth regulators vs. activated charcoal.

## 2. Results and Discussion

### 2.1. In Vitro Seed Germination

**Figure 1:**
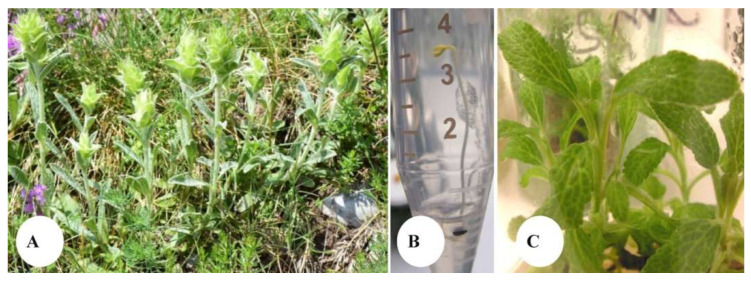
*Sideritis scardica* in its natural habitat on Slavyanka Mountain, Bulgaria (**A**), sterile germination in the SGM medium composition of the seeds collected from the wild habitat (**B**), and stock shoots of the planned maintainance in laboratory conditions (**C**).

In the experimental conditions described in this work, 0.65% of seed germination was recorded within two weeks after inoculation of the seeds collected from the wild growing plant (Figure 1A,B).

After one month of culture, germinated plantlets were transferred and further maintained as stock shoots in the C_0 culture medium (Figure 1C) and used as starting material for the tissue culture experiment.

### 2.2. Effect of In Vitro Culture Treatments on the Developmental Patterns of S. scardica

**Figure 2:**
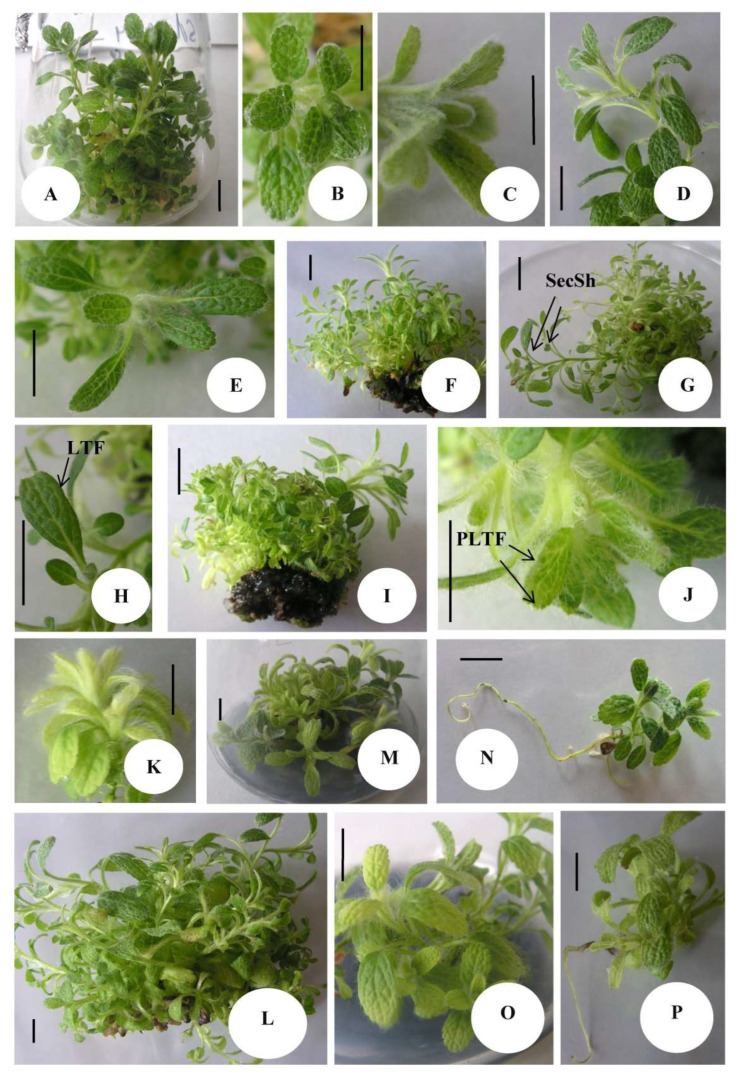
*Sideritis scardica* in shoot culture experimental conditions: shoot cultures (**A**) and leaf morphology in the PGR free C_0 (**B**), intensive trichomes formation in Sm (**C**); developed shoots in Sr_1 (**D**), Sr_2 (**E**,**F**); intensive secondary (SecSh) axillary shoots formation in 80% of primary shoots (**G**) and loss of teeth formation (LTF) on leaf rib margins of 65% of examined samples (**H**) in Sr_3, intensive callusogenesis (**I**) and loss of lateral and retained apical teeth formation on leaf margins (partial loss of teeth formation—PLTF) (**J**) in Sr_4; intensive trichome formation and increased IC in C_1 (**K**), intensive shoot formation in C_2 (**L**), inhibition of shoot length (**M**) and root formation in 40% of samples (**N**) in C_3, length suppression, trichomes stimulation (**O**), and root formation in 50% of samples in C_4 medium (**P**). Space bar in (**E**,**H**,**J**) = 0.5 cm and in all other photos = 1 cm.

Both types of treatments (PGRs and AC) were shown to result in a drop of shoot length in all experimented concentrations with the Sm treatment (0.2 mg/L benzyladenine, BA + 0.02 mg/L 1-naphthaleneacetic acid, NAA), giving the lowest value for this parameter (Table 1). Of all PGR treatments, the highest compactness of shoots was logically also established in the length-suppressed Sm medium modification. As for the AC treatments in all of them, with the exception of the 0.5 g/L AC supplementation (C_2), an increased IC, comparable to the Sm one, was established (Table 1, Figure 2K).

The number of axillary shoots was also generally stimulated in treated, as compared with control plants, with the highest value of the parameter observed in media where 0.5 mg/L BA was applied in combination with 0.5 or 1.0 mg/L NAA (Sr_3 and Sr_4, respectively). As for the AC treatments—0.05 g/L and 0.2 g/L (C_2 and C_3, respectively) led to the highest number of shoot multiplication. However, the type of axillary shoots formed differed when comparing the PGR and AC treatments (Figure 2). Thus, while the Sr_3 treatment (0.5 mg/L BA + 0.5 mg/L NAA) was characterized by an intensive secondary shoot formation in 80% of the primary shoots (Figure 2G), the intensively forming axillary shoots in C_2 medium (0.05 g/L AC) were primary shoots, developing from the explant base (Figure 2L). In addition, while lack of rooting in the PGR-free C_0 and callusogenesis were established in the PGR treated samples (Figure 2A–J), rooting was induced in 40% of samples in C_3 and 50% of samples in C_4 treatments (Figure 2N,P, respectively). Stimulation of trichome formation, as compared with PGR-free control plants, was established in the 0.2 mg/L BA + 0.02 mg/L NAA Sm treatment (Figure 2C) and more often in AC treatments (Figure 2K,O). Leaf morphology was also differentially affected by the two types of approaches with a total (Sr_3 treated samples, Figure 2H) or a partial (in Sr_4 treated samples, Figure 2J) loss of teeth formation on leaf ribs in the PGR supplemented culture media. Thus, as a general observation, in terms of multiplication intensity, application of activated charcoal was able to affect the developmental patterns of *S. scardica* shoot cultures in a manner similar to the one of PGR treatments. However, distinctive differences between the two approaches were observed, consisting of the higher stimulation of compactness of shoots, stimulation of rooting and leaf trichomes formation, as well as preservation of teething on leaf ribs in AC, as compared with auxin and cytokinin applied in this work.

The practical use of charcoal for the purification and the discoloration of liquids has been recorded since the 18th century [20]. Activation of charcoal by means of pyrolysis with carbon dioxide was achieved by Ostrejko in the beginning of the 20th century and led to a significant improvement of its adsorbing properties [21]. The huge area of activated charcoal (AC) ranges from 600 to 2000 m^2^ g/L, and pore distributions range from 10 to 500 µM [21]. Thus, AC is characterized by a huge inner area with a fine network of pores, capable of adsorbing many substances, resulting in the broad application of AC in micropropagation, orchid seed germination, somatic embryogenesis, anther culture, synthetic seed production, protoplast culture, rooting, stem elongation, bulb formation, etc. [20]. AC has also been applied in plant cell tissue and organ culture as a growth enhancing agent due to its capability of adsorbing excess secondary metabolites, leaking into the medium, or releasing potentially toxic physiological by-products, resulting from the tissue culture developmental processes of the plants [20,22]. Mechanisms, through which AC affects plant tissue culture development, have been systematized by Pan and Staden [20] in the following categories: (i) providing a dark environment—the darkening of the explant base has been hypothesized to lead to the enhanced accumulation of photosensitive auxin or other co-factors. This suggestion has been supported by examples of enhanced root formation in Allium cepa [23], Diuris Iongifolia [24], Glycine max [25], etc.; (ii) Adsorption of undesirable or inhibitory substances in the in in vitro cultures—the authors have pointed out the advantage of AC application for adsorption of excessive phenolic build-up, as compared with the frequent transfer of explants to fresh medium (the latter leading to an increased risk of mutations occurrence and loss of embryogenic potential). Thus, for example, AC was able to reduce browning and to enhance survival and morphogenic response, while PVP, ascorbic acid, or dihydroxynaphathalene failed to provide a satisfactory effect on palm explants in tissue culture conditions [26]; (iii) Adsorption of plant growth regulators in the in vitro culture—Pan and Staden [20] have summarized the ability of AC to adsorb not only exogenously supplemented to the solid or liquid culture media auxins and cytokinins, but also the potential of this additive to control the excessive release of abscisic acid and ethylene released by the in vitro cultivated plant tissues; (iv) Substances released from charcoal—the investigations on the potential role of AC to contribute to the mineral content of tissue culture media as have started as early as 1975 [27]; (v) Other effects have also been shown to underline the influence of AC on plant tissue culture development. Thus, AC has been shown to have an effect on sucrose hydrolysis during autoclaving, this effect being related to the pH of the culture medium [28,29]. Today, active research is still ongoing on the favorable effects of AC application in many aspects of plant cell tissue and organ culture. Thus, AC has been shown to effectively enhance in vitro seed germination of the endangered medicinal plant *Nothapodytes foetida,* when applied in combination with benzylaminopurine and gibberellic acid [30]. An improvement of the micropropagation protocol of red-fleshed *Hylocereus* species (Cactaceae) has been achieved through combining AC with 1-naphthaleneacetic acid and 6-benzylaminopurine [31].

A recent work by Dong et al. [32] sheds light on the molecular aspects of the AC-stimulated germination of wheat seedlings. It was established that 9460 differentially expressed genes (DEGs) were upregulated, and 7483 DEGs were downregulated in the presence of AC, as compared to the untreated seedlings. The authors elucidated that AC stimulated the expression of genes of the phenylpropanoid biosynthesis pathway, whose protein products promote cell differentiation and seedling growth. Interestingly, while genes involved in plant hormone signaling related to stress resistance and disease resistance were also upregulated, the genes related to plant growth inhibition were downregulated.

Thus, although a piling number of scientific reports have been focused on the broad application of AC on plant tissue culture morphogenesis, information on the effect of this treatment on secondary metabolite production is practically missing.

### 2.3. Effect of PGRs and AC on 5-Caffeoylquinic Acid Content In Vitro and In Situ

**Figure 3:**
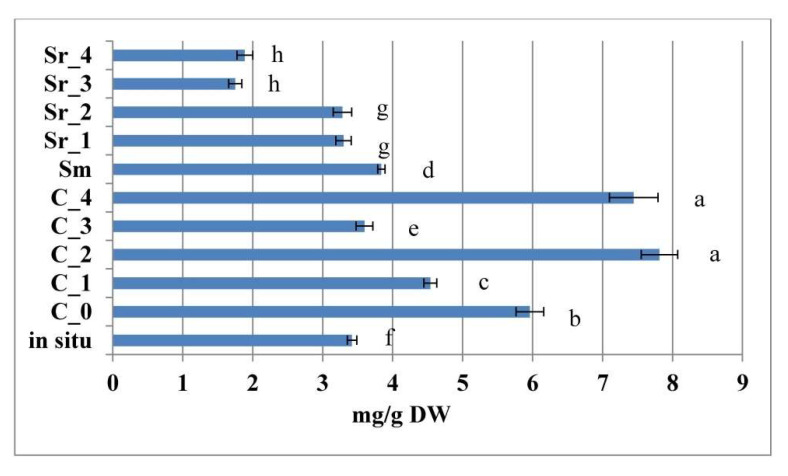
5-Caffeoylquinic acid content in AC and PGR treated, as well as in situ collected samples of *Sideritis scardica*. Error bars represent SEM. Means followed by the same letter do not differ statistically at *p* ≤ 0.05 according to the Tukey test.

As a general observation, all treatment types (with the exception of Sr_3 and Sr_4, 0.5 mg/L BA + 0.5 mg/L or 1.0 mg/L NAA, respectively) led to the accumulation of comparable or elevated levels of 5-caffeoylquinic acid of in vitro cultivated plants as compared with in situ collected plant material (Figure 3).

When comparing the different types of treatment approaches, it was established that the highest levels were obtained for the PGR-free C_0 control and the C_1, C_2, and C_4 AC treatments.

### 2.4. Effect of PGRs and AC on Phenylethanoid Glycosides In Vitro

**Figure 4:**
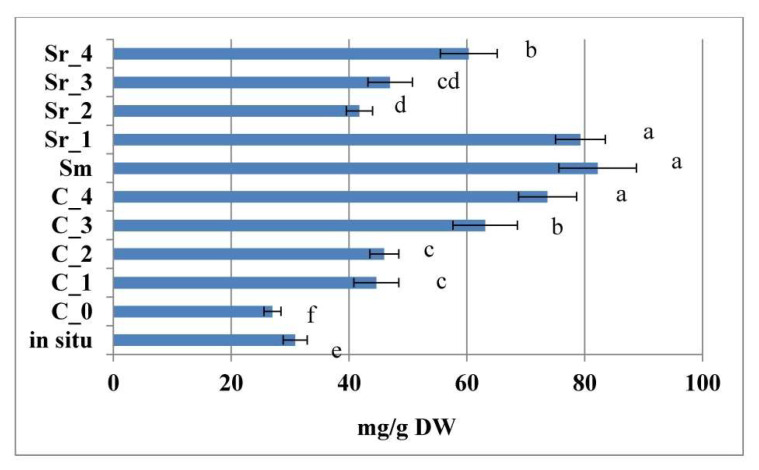
Sum of the total phenylethanoid content in AC- and PGR-treated samples, as well as in situ collected samples of *Sideritis scardica*. Error bars represent SEM. Means followed by the same letter do not differ statistically at *p* ≤ 0.05, according to the Tukey test.

The content of total phenylethanoid glycosides in samples is presented in Figure 4, and the content of individual phenylethanoids in samples is presented in Table S1. The chromatograms of representatives of each treatment group (in situ, PGR-treated, and AC) are presented in Figure S1.

Phenylethanoid glycosides are a group of phenolic compounds, encompassing hydroxyphenylethyl alcohol, hydroxycinnamic acids, and glycosyl moieties. The pioneering research on PhGs was performed by the isolation and characterization of echinacoside from *Echinacea angustifolia* (Asteraceae) in 1950 and verbascoside from *Verbascum sinuatum* (Scrophulariaceae) in 1963 [33]. The phenylethanoid glycoside verbascoside (acteoside) is a chemotaxonomic marker compound of the order Lamiales [34]. Some Lamiales plants also contain (i) isomers of verbascoside, such as isoacteoside, forsythoside A, forsythoside H, and forsythoside I, whose rhamnosyl and caffeoyl units are linked at different glucosyl positions, and (ii) closely related compounds of verbascoside, such as plantamajoside and isoplantamajoside, which contain a glucosyl unit instead of the rhamnosyl units [35].

While the in situ collected samples of the plant were characterized by a slight domination of lavandulifolioside and verbascoside over the rest of detected components (echinacoside, echinacoside isomer, forsythoside B, and lowest levels of forsythoside A and leucoseptoside A, Table S1), the tissue culture development of the species resulted in alteration of the dominant individual components observed in this work. Thus, while in the PGR-free C_0 treatment, verbascoside was the dominating component in levels, significantly exceeding the ones of the wild plant, with further treatments leading to even higher stimulation of the component with highest levels—observed in the C_2, C_3, and C_4, as well as in the Sm media modifications (Table S1). Verbascoside levels were the lowest of all samples in the Sr_2 treatment. Higher AC concentrations, as well as the PGR treatments, led to a marked stimulation of echinacoside, as compared with the wild-collected and PGR-free cultivated plant. Lavandulifolioside levels were also stimulated by treatments, as compared with the PGR-free control, with levels also exceeding the ones of the wild samples (with the exception of Sr_2 and Sr_3 treatments). Tissue culture development (in C_0, C_1, C_2, C_4, and Sr_3) led to the detection of isoleucoseptoside, which was not detected in the wild samples. The Sm and Sr_3 treatments also led to the induction of leucoseptoside glycoside, which was also not detected in the wild samples. Thus, by generalizing the above data, by comparing the total sum of the phenylethanoid components in the samples (Figure 4), it was established that both treatment approaches stimulated this parameter as compared with the PGR-free control and the wild collected plant.

### 2.5. Effect of PGRs and AC on Flavone Glycosides In Vitro

**Figure 5:**
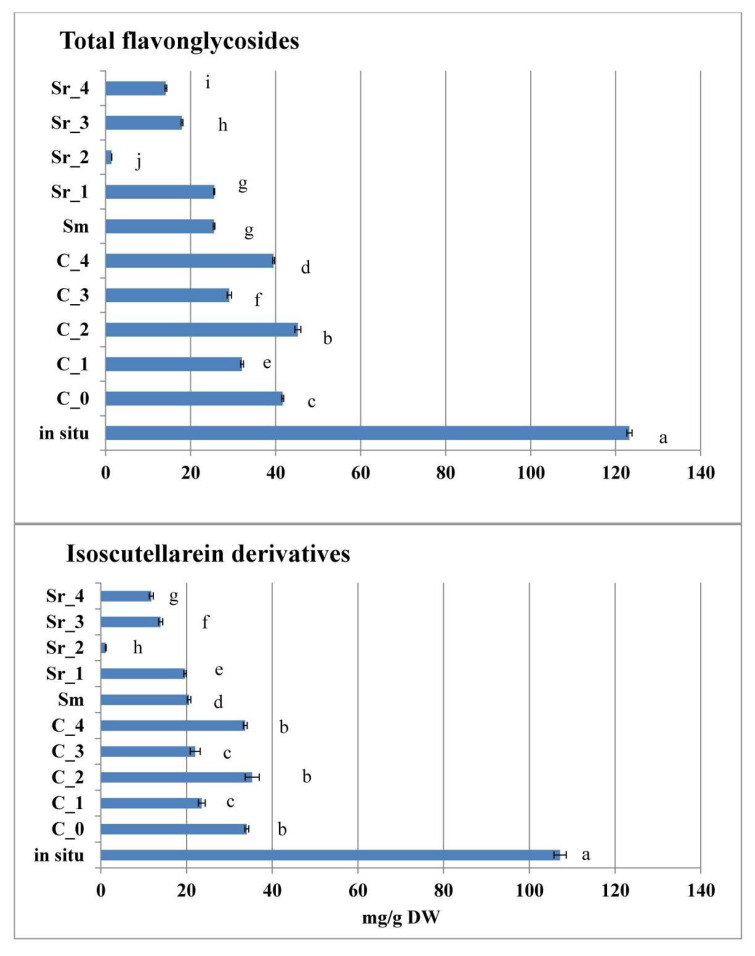
Isoscutellarein derivatives as the dominants group in total flavone-glycosides content in AC and PGR treated, as well as in situ collected samples of *Sideritis scardica*. Error bars represent SEM. Means followed by the same letter do not differ statistically at *p* ≤ 0.05 according to the Tukey test when comparing one and the same parameter for all samples.

Isoscutellarein derivatives were the dominant flavone-glycosides group, contributing to the total pool of these metabolites in all experimental samples (Figure 5, Table S2).

**Figure 6:**
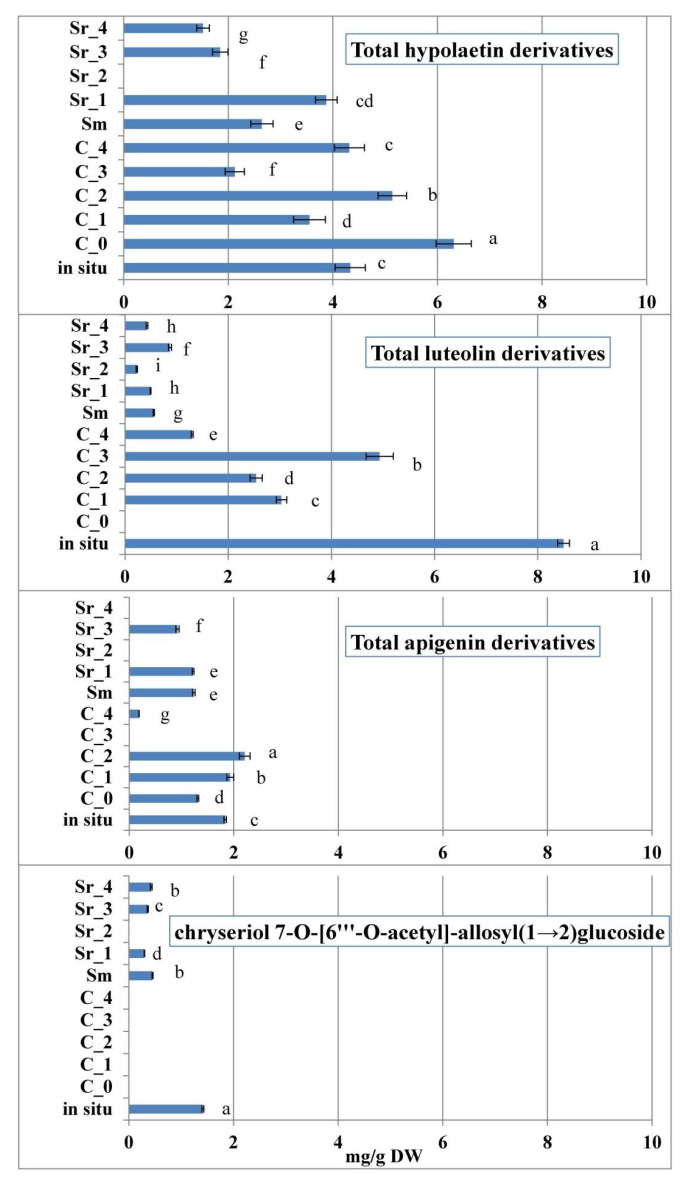
Other flavone glycoside derivatives, contributing to the total pool of these metabolites in AC and PGR treated, as well as in situ collected samples of *Sideritis scardica.* Error bars represent SEM. Means followed by the same letter do not differ statistically at *p* ≤ 0.05 according to the Tukey test when comparing one and the same parameter for all samples.

Other derivatives, contributing to this phytochemical group, were hypolaetin, luteolin, and apigenin derivatives, as well as chrysoeriol 7-*O*-[6′′′-*O*-acetyl]-allosyl(1→2)glucoside, which was identified as the sole chrysoeriol in the present study (Figure 6, Table S2). The dominant isoscutellarein derivative in *S. scardica* samples was isoscutellarein 7-*O*-[6′′′-*O*-acetyl]-allosyl(1→2)glucoside, followed by isoscutellarein 7-*O*-[6′′′-*O*-acetyl]-allosyl(1→2)-[6″-*O*-acetyl]-glycoside (Table S2). For both of these compounds, in vitro culture development led to a drop of accumulated levels, as compared with the wild collected samples. When comparing the two types of in vitro treatments, AC significantly stimulated the accumulation of these two isoscutellarein derivatives, as compared with PGR treatments. The latter tendency also applied for 4′-*O*-methylisoscutellarein 7-*O*-[6′′′-*O*-acetyl]-allosyl(1→2)glucoside, whose amount followed third in ranking in the total isoscutellarein pool. The second group of flavone glycoside derivatives, according to their accumulation levels, was the luteoline derivatives, followed by the hypolaetin, apigenin, and chrysoeriol ones (Figure 6, Table S2).

Luteolin 7-*O*-allosyl(1→2)glucoside was the dominant luteoline derivative, with the highest levels established in the wild collected material. Interestingly, this compound was not detected in the PGR-free control and PGR-treated plants (with the exception of low levels in Sr_3), but it was detected in the AC C_1–C_4 media modifications. The second most abundant derivative of this group was luteolin 7-*O*-allosyl(1→2)-[6″-*O*-acetyl]-glucoside, which was only detected in wild collected plants. It was followed as accumulation levels by luteolin 7-*O*-[6′′′-*O*-acetyl]-allosyl(1→2)-glucoside, and it was also found only in wild collected samples. On the other hand, methyl-luteolin 7-*O*-allosyl(1→2)glucoside was not found in wild collected and PGR-free samples, but only in AC treated plants, as higher levels were established in the C_1–C_4 treatments. The dominant component of the hypolaetin derivatives group was 3′-*O*-methylhypolaetin 7-*O*-[6′′′-*O*-acetyl]-allosyl(1→2)glucoside, with commensurable levels in the wild collected, PGR-free, and AC-treated *S. scardica* samples (Table S1). Accumulation level of this compound was followed by the one of 4′-*O*-methylhypolaetin 7-*O*-[6′′′-*O*-acetyl]-allosyl(1→2)-[6″-*O*-acetyl]-glucoside, which was not detected in the wild collected samples, and it was accumulated predominantly in the PGR-free and AC (with the exception of C_3) samples. Of the PGR-treated *S. scardica,* this component was only detected in Sm and Sr_1. Of all samples, 3′-*O*-methylhypolaetin 7-*O*-allosyl (1→2)glucoside was only detected in the C_0 PGR-free control plants. Hypolaetin 7-*O*-[6′′′-*O*-acetyl]-allosyl(1→2)glucoside was only detected in wild collected, Sm, and Sr_1 treated plants. The most abundant of apigenin derivatives—apigenin glucoside—was detected in the highest amount in the wild collected samples, and it was produced in significantly lower levels in C_0–C_2, as well as in Sm, Sr_1, and Sr_3 tretaments. Apigenin 7-*O*-[6′′′-*O*-acetyl]-allosyl(1→2)glucoside was produced in higher levels in situ, followed by C_0—C_2, as well as Sm, Sr_1, and Sr_3 treatments. Apigenin 7-(4″-*p*-coumaroylglucoside) was not detected in situ, and it was produced in C_0, C_1, C_2, and C_4, as well as Sm and Sr_1 treatments. Of all studied samples, apigenin-7-O-glucoside was detected only in C_1 and C_2 treatments. Chrysoeriol 7-*O*-[6′′′-*O*-acetyl]-allosyl(1→2)glucoside was mainly produced in wild collected samples, and it was produced in lower amounts in PGR-treated ones (with the exception of Sr_2). Thus, as a general observation, the in situ plant material was shown to accumulate the highest total flavone-glycoside levels, as compared with tissue culture-derived *S. scardica*. When comparing between the two in vitro culture approaches, however, the PGR-free control plants and the AC-treated plants were generally higher producers of flavone-glycosides, as compared with PGR-treated plants (Figure 6). As discussed above, five of the flavone glycoside derivatives were detected only in tissue culture-derived plant material and absent in the wild collected one.

**Figure 7:**
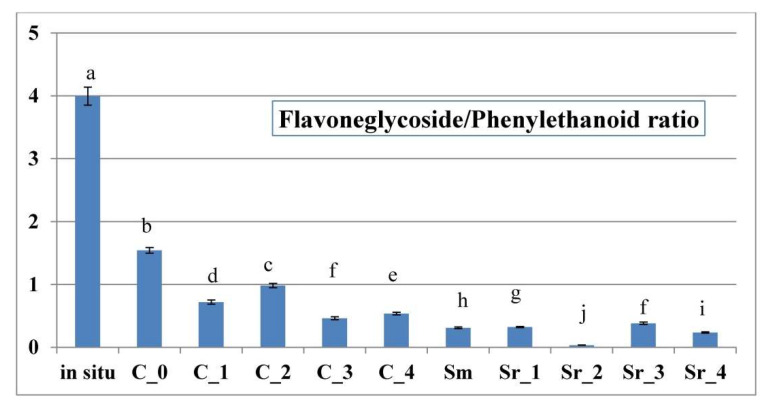
Ratio of total pools of flavone glycoside/phenylethanoide derivatives (F/P) in AC- and PGR-treated samples, as well as in situ collected samples of *Sideritis scardica.* Error bars represent SEM. Means followed by the same letter do not differ statistically at *p* ≤ 0.05 according to the Tukey test.

It was established that, while in the wild collected, control, and AC *S. scardica* samples, flavone glycosides dominated over phenylethanoides, the opposite ratio was observed in PGR-treated *S. scardica* plants (Figure 7). When comparing the two distinctive tissue culture approaches applied in the current work, AC was shown to favor flavone glycoside biogenesis, as compared with PGR treatments (expressed as the elevation of the flavone glycoside/phenylethanoid ratio, Figure 7).

Studies on phenylethanoid biogenesis were performed by Ellis [36] in a model system of lilac suspension culture, which produced phenylethanoid glycosides in amounts up to 16% of their dry weight. Verbascoside (as the main component) and salidroside were obtained in this experimental system. The study demonstrated that the phenylacrylic acid of the two compounds was partially derived from phenylalanine or cinnamic acid, and the hydroxyphenyl ethyl group was derived partially from tyrosine or tyramine. Due to its complexity, the biogenesis of phenylethanoids remains still to be elucidated. In a recent study, Yang et al. [37] shed light on the subject by the identification of two of the missing enzymes of the verbascoside pathway, namely, hydroxycinnamoyl-CoA:salidroside hydroxycinnamoyltransferase (SHCT), involved in region-selective acylation of salidroside to form osmanthuside A, and osmanthuside B 3,30-hydroxylase (OBH)—responsible for the catalyzation of meta-hydroxylations of the *p*-coumaroyl and tyrosol moieties of osmanthuside B in the final steps of verbascoside biosynthesis. Tissue culture models have been utilized to perform functional experiments of elucidation of the effects of different environmental treatments on phenylethanoid accumulation in vitro. In an experiment with a callus culture of *Cistanche salsa* (C.A.Mey.) (Beck), it was shown that the accumulation of echinacoside, acteoside, and 2′-acetylacteoside were dependent on the carbon source, auxin, and culture conditions [38]. The modification of hormone ratio and light conditions led to the stimulation of phenylethanoid glycosides in this model system. Abiotic stress elicitors, such as methyl jasmonate and salicylic acid, were also shown to stimulate phenylethanoid production in *Cistanche deserticola* Ma suspension [39]. Phenylethanoid stimulation in a *S. scardica* in vitro model system in the current work was clearly enhanced in PGR treatments, which were related to morphological and physiological disturbances of the plant growth and development, while flavone glycosides were favored in the in situ collected, PGR-free control, and AC-treated plants.

### 2.6. Principal Component Analysis of Obtained Data of the Phytochemical Studies

**Figure 8:**
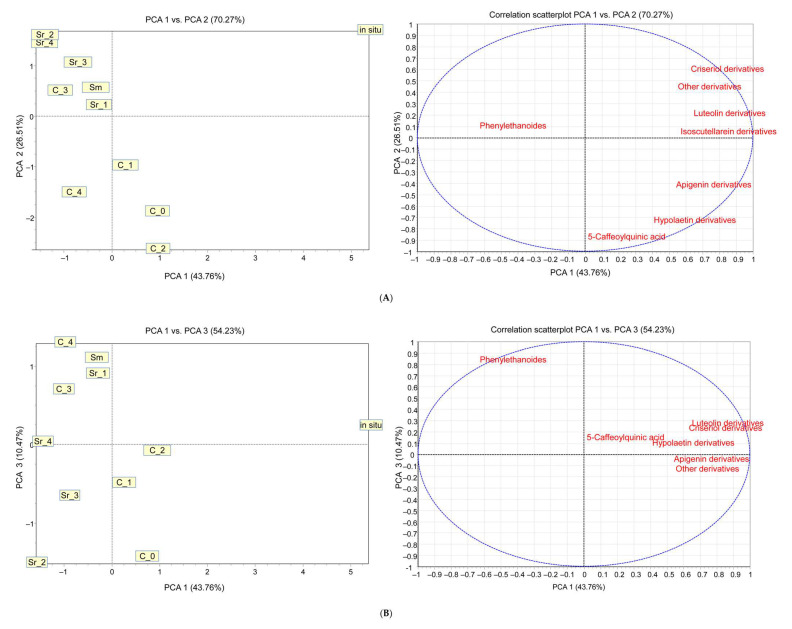
Principal component analysis score plot and correlation scatterplot of the variables with (**A**) PC1 and PC2 and (**B**) PC1 and PC3, based on 5-caffeoylquinic acid, total luteolin, chrysoeriol, isoscutellarein. Hypolaetin, and phenylethanoid derivatives. For codes, see Table S3.

In order to evaluate the significance of the variables’ effect of in vitro culture treatments on phytochemical composition, principal component analysis (PCA) was applied, and eight principal components were obtained. The first factor (PC1), which explained 43.76% of the variance, was mainly associated with luteolin, chrysoeriol, and isoscutellarein derivatives. The second principal component (PC2), which explained 26.51% of the total variance, was related to 5-caffeoylquinic acid and hypolaetin derivatives, whereas the third factor (PC3), contributing 10.47% to the total variance, was related to phenylethanoid derivatives. The principal component score plot and correlation scatterplot of the variables with PC1 and PC2 and PC1 and PC3, based on 5-caffeoylquinic acid, total luteolin, chrysoeriol, isoscutellarein. Hypolaetin, and phenylethanoid derivatives, are presented in Figure 8A,B. The eigen values and factor loadings from PCA are provided in Table S3.

According to all factors, a significant difference can be found between in situ and all other samples due mainly to the high content of isoscutellarein and also luteolin and chrysoeriol derivatives. The first factor, PCA1, distinguishes the AC (C_1-C_4) and PGR (Sm, Sr_1-Sr_4)-treated samples. AC treated samples produce more isoscutellarein and luteolin derivatives than the others, and they do not produce chrysoeriol derivatives at all. The difference in the second factor can be found according to 5-caffeoylquinic acid and hypoletin derivatives. Their content is slightly higher in AC-treated samples than in PGR-treated samples, but both were in a similar range of the control and in situ. The third factor is correlated with the content of phenylethanoid derivatives. Similarity can be found between both group of treated samples, which is higher than the control sample (C_0) and in situ.

## 3. Materials and Methods

### 3.1. Plant Material

Seeds of the wild growing plant were collected at the Shabran peak, Slavyanka Mountain, Bulgaria (Figure 1A). For the purpose of extraction, aerial parts of *S. scardica* were collected at the flowering stage. The air drying of the plant material was performed in the shade, at room temperature. Prior to analyses, the material was stored in a desiccator and processed, as described below, within a period three months after its collection.

### 3.2. Tissue Culture Initiation

For the purpose of in vitro germination, seeds were surface-sterilized with 30 s of 70% ethanol (puriss. p. a. absolute, (GC), Sigma-Aldrich, St. Louis, MO, USA) immersion, followed by 7 min of 0.1% HgCl_2_ (Mercury(II) chloride, 98+%, Alfa Aesar, Haverhill, MA, USA) treatment and triplicate rinse in sterile distilled water. Afterwards, seeds were dried on sterile filter paper. For germination, seeds were then inoculated on germination medium with the following composition: half-strength macro-salts Murashige and Skoog culture medium [40] supplemented with Gamborg vitamins [41], 2 mg/L glycine, 20 g/L sucrose, and 6.0 g/L agar (for microbiology, Sigma-Aldrich), and they were placed in the dark, at 25 ± 1 °C. The axillary shoots obtained after germination (Figure 1B) were further maintained on plant growth regulators (PGR)—free Murashige and Skoog macro- and microsalts medium with Gamborg vitamins and 2 mg/L glycine supplementation, 30 g/L sucrose, and 6.5 g/L agar (C_0 medium), which occurred at 25 ± 1 °C at a 16/8 h photoperiod and for 8 weeks of regular subculture (Figure 1C).

### 3.3. Tissue Culture Experiment

The effect of PGRs on the in vitro cultivated plant was experimented by the following treatments: the PGR-free C_0, as described above, supplemented with 0.2 mg/L BA and 0.02 mg/L NAA (Sm), 0.2 mg/L BA and 0.5 mg/L NAA (Sr_1), 0.2 mg/L BA and 1.0 mg/L NAA (Sr_2), 0.5 mg/L BA and 0.5 mg/L NAA (Sr_3), and 0.5 mg/L BA and 1.0 mg/L NAA (Sr_4). For the purpose of AC treatment, the following media were experimented: 0.02 g/L AC (C_1), 0.05 g/L AC (C_2), 0.2 g/L AC (C_3), and 0.5 g/L AC (C_4) (Table 2). Plant growth regulators and AC were purchased by Duchefa Biochemie: (BA)—6-benzylaminopurine (6-BAP), CAS number 1214-39-7, Assay > 99%; (NAA)—α-naphtalene acetic acid, CAS number 86-87-3, Assay > 98%); (AC)—Charcoal Activated—CAS number 7440-44-0, Assay > 100%, pH (5% in water) 5–7.

Five explants were inoculated in a culture vessel of 100 mL culture medium. Five culture vessels of each treatment were cultivated for each set of biological replications. Plant material of two biological replications was collected. Cultures were kept at 25 ± 1 °C at a 16/8 h photoperiod (Daylight Fluorescent Tube, Philips TL-D S80 865, irradiation intensity of 60 μmol/m^2^ per second) and 8 weeks of regular subculture, and the results were recorded after 12 weeks of growth. The number of leaf couples per each shoot were counted, and the index of compactness (IC) was calculated as the number of leaf couples per cm for each respective shoot.

### 3.4. Extraction of Plant Material

For the purpose of the phytochemical analyses, 0.2 g of the dry plant material of wild-collected and in vitro cultivated plants was extracted with 70% MeOH (puriss. p.a. ACS reagent, ≥99.8% (GC)) for 30 min in ultrasonic bath (Elmasonic S 30 (H) at ultrasonic frequency 37 kHz) at room temperature. The obtained extracts were evaporated in vacuum at 40 °C, kept in a desiccator till constant weight, and stored at −18 °C. Prior to analyses, dry extracts were dissolved in 70% methanol. Analyses were made in triplicate.

### 3.5. LC/MS Analyses

Chromatographic separations were performed on a 250 mm × 4.6 mm, 5 µm C18 Luna column (Phenomenex, Madrid Avenue Torrance, CA 90501-1430, USA). The mobile phase consisted of two solvents: water—formic acid (1%, *v*/*v*) and methanol, as solvents A and B, respectively. A linear gradient with 35% B was used at the first 5 min, increasing linearly to 50% B to 30 min, 100% B at 35 min, and 100% B from 35 to 55 min. The flow rate was 0.5 mL/min. The injection volume was 20 µL.

The HPLC system was an Agilent series 1100 equipped with a binary pump, diode array detector, and ion trap mass detector in series (Agilent Technologies, Waldbronn, Germany), controlled by the ChemStation software (Agilent, v.08.03).

Spectral data from all peaks were accumulated in the range 190–600 nm, and chromatograms were recorded at 290 and 300 nm for flavonoid derivatives and at 330 nm for phenylethanoid glycosides and hydroxycinnamic acids.

The mass detector was an Ion-Trap Mass Spectrometer equipped with an electrospray ionisation (ESI) system and controlled by LCMSD software (Agilent, v.6.1.). Nitrogen was used as a nebulising gas at a pressure of 65 psi, and the flow was adjusted to 12 L/min. The heated capillary and the voltage were maintained at 325 °C and 4 kV, respectively. MS data were acquired in the negative ionization mode. The full scan covered the mass range at *m*/*z* 100–1200. Collision-induced fragmentation experiments were performed in the ion trap using helium as collision gas, with a voltage ramping cycle from 0.3 up to 2 V. Maximum accumulation time of the ion trap and the number of MS repetitions to obtain the MS average spectra were set at 300 ms and 5, respectively.

### 3.6. Identification and Quantification of Phenolic Compounds

The identification and peak assignment of all phenolic compounds was based on a comparison of their retention times and UV absorption and mass spectral data with those of the standards and the published data [13,42,43].

Hydroxycinnamic acids were quantified using 5-caffeoylquinic acid external standard at 330 nm, phenylethanoid glycosides were quantified and expressed as verbascoside equivalent at 330 nm, hypolaetin glycosides were quantified with 4′-*O*-methylhypolaetin 7-*O*-[6′′′-*O*-acetyl]-allosyl(1→2)glucoside at 290 nm, whereas isoscutellarein, luteolin, and apigenin glycosides were quantified and expressed as 4′-*O*-methylisoscutellarein 7-*O*-[6′′′-*O*-acetyl]-allosyl(1→2)glucoside equivalent at 300 nm. The stock solutions of phenolic standards were made up in 70% methanol to a concentration of 1 mmol/L. The corresponding calibration curves were constructed with five dilutions of the stock solutions.

### 3.7. Statistical Analyses

Statistical analysis of the data of the phytochemical analyses was performed using Excel 2017 for calculations of calibration curves, mean, standard deviation, and standard error of the means. All values reported in this work are means ± SE (standard error of the mean), and the exact numbers of the experiments are present at the respective sub-sections of the Material and Methods section. The significance of differences between the treatments for one and the same parameter was analyzed at *p* ≤ 0.05 by a post hoc Tukey-HDS test after performing ANOVA single factor analysis with the help of the Real Statistics Resource Pack on Excel 2019. Principal component analysis was performed using the software TANAGRA 1.4.28 (Lyon, France).

## 4. Conclusions

The experimental results of the present work led to two principal observations related to *S. scardica* biosynthetic potential. On one hand, tissue culture development resulted in identification of two phenylethanoids and five flavone glycosides, not detected in the wild collected plant material. On the other hand, two principally different tissue culture approaches showed the plasticity of the capacity of this species to produce phenolic compounds—with AC treatment stimulating flavone glycosides production (similarly to the wild collected plant), while PGRs favored the production of phenylethanoid derivatives.

## Figures and Tables

**Table 1:** Effect of PGRs and AC on the morphometry of *S. scardica* in vitro.

Medium Abbreviation	Shoot Length, cm	IC, Leaf Couples per cm	Number Axillary Shoots
C_0	5.90 ± 0.50 ^a^	1.40 ± 0.05 ^f^	9.00 ± 0.80 ^f^
Sm	1.83 ± 0.05 ^f^	3.21 ± 0.05 ^a^	8.67 ± 0.69 ^f^
Sr_1	3.90 ± 0.20 ^bc^	1.70 ± 0.10 ^d^	10.00 ± 0.80 ^ef^
Sr_2	4.00 ± 0.17 ^bc^	1.60 ± 0.07 ^de^	16.50 ± 0.70 ^d^
Sr_3 *	3.40 ± 0.51 ^cd^	1.50 ± 0.09 ^e^	35 ± 1.20 ^a^
Sr_4	3.00 ± 1.50 ^bcd^	1.75 ± 0.50 ^de^	25 ± 1.50 ^c^
C_1	2.90 ± 0.10 ^d^	2.90 ± 0.20 ^b^	10 ± 0.90 ^e^
C_2	4.30 ± 0.30 ^b^	1.60 ± 0.04 ^d^	32 ± 1.30 ^b^
C_3 **	2.50 ± 0.30 ^e^	3.10 ± 0.30 ^ab^	15 ± 1.20 ^d^
C_4 ***	3.83 ± 0.50 ^bc^	2.47 ± 0.10 ^c^	9 ± 0.90 ^e^

* Formation of secondary axillary shoots up to 1.5 ± 0.3 cm in 80% of primary shoots, ** Root formation in 40% of samples (root length 4.5 ± 1), *** Root formation in 50% of samples (root length 2.5 ± 0.8), ±values represent SEM. Means followed by the same letter do not differ statistically at *p* ≤ 0.05, according to the Tukey test, when comparing one and the same parameter for all treatments.

**Table 2:** Plant growth regulators (PGR) and activated charcoal (AC) supplementations for the purpose of a tissue culture experiment with *S. scardica* shoot cultures.

Medium Abbreviation	PGR Supplementation	Medium Abbreviation	AC Supplementation
C_0	PGR-free	C_0	AC-free
Sm	0.2 mg/L BA + 0.02 mg/L NAA	C_1	0.02 g/L
Sr_1	0.2 mg/L BA + 0.5 mg/L NAA	C_2	0.05 g/L
Sr_2	0.2 mg/L BA + 1.0 mg/L NAA	C_3	0.2 g/L
Sr_3	0.5 mg/L BA + 0.5 mg/L NAA	C_4	0.5 g/L
Sr_4	0.5 mg/L BA + 1.0 mg/L NAA		

## Data Availability

Data are available from the corresponding co-authors upon request.

## Supplementary Materials

The following supporting information can be downloaded at: https://www.mdpi.com/article/10.3390/plants12132541/s1, Figure S1: HPLC-DAD chromatograms at 330 nm for in situ sample, PGR supplementation (Sr_3) and AC supplementation (C_1); Table S1: HPLC-UV/Vis-ESI-MS/MS data and individual phenylethanoids content (mg/g, *n* = 3) in AC and PGR treated, as well as in situ collected samples of *Sideritis scardica*; Table S2: HPLC-UV/Vis-ESI-MS/MS data and individual flavone glycosides content (mg/g, *n* = 3) in AC and PGR treated, as well as in situ collected samples of *Sideritis scardica*; Table S3: (a) Eigen values from PCA analysis.
