# Supplementary material for: Plant Growth Regulators and Activated Charcoal Selectively Affect Phenylethanoid and Flavone Glycoside Accumulation in Sideritis scardica Griseb. Tissue Culture
Source: Plants (Basel). 2023 Jul 4;12(13):2541. doi: 10.3390/plants12132541 (PMC10346976; doi:10.3390/plants12132541)
Supplement: Supplementary file 1 [file plants-12-02541-s001.zip › plants-2446918-supplementary.pdf]

Table S1. HPLC-UV/Vis-ESI-MS/MS data and individual phenylethanoids content (mg/g, *n*=3) in AC and PGR treated, as well as in situ collected samples of *Sideritis scardica*

	t_R	UV max	$[M-H]^-$	MS^2	*In situ*	C_0	C_1	C_2	C_3	C_4	Sm	Sr_1	Sr_2	Sr_3	Sr_4
5-Caffeoylquinic acid	9.7	242, 294sh, 326	353	191, 179	3.42	5.96	4.54	7.82	3.60	7.45	3.84	3.30	3.28	1.75	1.89
Echinacoside	12.6	232, 246sh, 290, 332	785	623, 461	3.77	4.32	11.89	10.96	22.07	31.76	30.20	35.02	19.07	18.91	26.29
Lavandulifolioside	12.9	232, 246, 290, 332	755	623, 593, 461	7.26	3.91	12.99	8.66	19.38	8.32	13.66	13.54	6.42	6.67	8.99
Verbascoside	13.6	232, 244, 290, 302sh, 332	623	487, 477, 461, 443, 315, 297	8.65	15.07	16.53	22.36	19.82	19.90	20.14	15.67	4.50	10.59	11.68
Forsythoside A	13.9	246, 286, 304, 334	623	578, 463, 461, 445, 316, 301	1.72	nd	nd	nd	nd	nd	nd	0.46	0.90	1.02	1.26
Leucoseptoside glycoside	15.7	234, 292sh, 328	799	637, 623, 461	nd	nd	nd	nd	nd	nd	1.41	nd	nd	0.32	nd
Echinacoside isomer	15.9	232, 246sh, 290, 332	785	623, 461, 315	3.36	nd	nd	nd	nd	4.15	11.74	3.25	3.11	2.17	4.48
Forsythoside B	16.0	232, 246, 290, 332	755	623, 593, 461, 315	4.59	nd	nd	nd	nd	5.32	5.06	7.49	5.98	4.62	4.31
Leucoseptoside A	16.8	234, 288, 330	637	491, 461, 443, 325	1.51	2.08	2.49	3.21	1.84	3.62	nd	3.27	1.77	2.67	3.33
Isoleucoseptoside	17.9	234, 288, 330	637	491, 461, 443, 325	nd	1.63	0.74	0.79	nd	0.62	nd	0.57	nd	nd	nd

nd – not detected

Table S2. HPLC-UV/Vis-ESI-MS/MS data and individual flavone glycosides content (mg/g, *n*=3) in AC and PGR treated, as well as in situ collected samples of *Sideritis scardica*

	t_R	UV max	[M-H]$^-$	MS^2	in situ	C_0	C_1	C_2	C_3	C_4	Sm	Sr_1	Sr_2	Sr_3	Sr_4
**Luteolin derivatives**															
Luteolin 7-*O*-allosyl(1→2)-[6″ -*O*-acetyl]-glucoside	17.9	256, 350	651	609, 447, 285	2.78	nd	nd	nd	nd	nd	nd	nd	nd	nd	nd
Luteolin 7-*O*-allosyl(1→2)glucoside	18.6	256, 352	609	591, 447, 429, 285	4.79	nd	2.54	1.79	4.36	0.66	nd	nd	nd	0.41	nd
Luteolin 7-*O*-[6‴-*O*-acetyl]-allosyl(1→2)-glucoside	20.3	256, 350	651	591, 429, 285	0.93	nd	nd	nd	nd	nd	nd	nd	nd	nd	nd
Methyl-luteolin 7-*O*-allosyl(1→2)glucoside	33.7	256, 352	623	461, 443, 299	nd	nd	0.49	0.76	0.57	0.64	0.56	0.49	0.23	0.46	0.43
**Apigenin dervatives**															
apigenin glucoside	21.2	266, 290, 332	431	269	1.03	0.25	0.20	0.23	nd	nd	0.33	0.25	nd	0.35	nd
apigenin 7-*O*-[6‴-*O*-acetyl]-allosyl(1→2)glucoside	21.6	266, 290sh, 344	635	593, 431, 413, 269	0.81	0.63	0.52	0.68	nd	nd	0.71	0.62	nd	0.58	nd
apigenin 7-(4″-*p*-coumaroylglucoside)	35.4	232, 268, 318	577	431, 413, 307, 269	nd	0.43	0.41	0.49	nd	0.19	0.20	0.35	nd	nd	nd

apigenin-7-*O*-glucoside	39.2	266, 290, 332	577	269	nd	nd	0.81	0.81	nd	nd	nd	nd	nd	nd	nd
**chryseriol derivatives**															
chryseriol 7-*O*-[6‴-*O*-acetyl]-allosyl(1→2)glucoside	22.3	252, 270, 346	665	623, 503, 461, 443, 299, 284	1.41	nd	nd	nd	nd	nd	0.45	0.30	nd	0.36	0.43
**Isoscutellarein derivatives**															
Isoscutellarein 7-*O*-[6‴-*O*-acetyl]-allosyl(1→2)glucoside	23.7	230, 276, 306, 330	651	609, 591, 447, 429, 285	63.22	21.33	16.45	24.51	14.18	20.01	13.09	12.19	0.64	7.98	7.02
4′-*O*-Methylisoscutellarein 7-*O*-allosyl(1→2)glucoside	28.4	230, 292, 314	623	461, 443, 299	2.09	nd	nd	nd	1.44	nd	0.32	0.29	nd	0.33	0.28
Isoscutellarein 7-*O*-allosyl(1→2)-[6″-*O*-acetyl]-glucoside	30.6		651	471, 285	1.41	nd	nd	nd	nd	nd	nd	nd	nd	0.30	0.23
4′*O*-Methylisoscutellarein 7-*O*-allosyl(1→2)-[6″-*O*-acetyl]-glucoside	33.9	230, 276, 306, 328	665	299	1.50	nd	nd	nd	nd	nd	nd	nd	nd	nd	nd
4′-*O*-Methylisoscutellarein 7-*O*-[6‴-*O*-acetyl]-allosyl(1→2)glucoside	36.7	230, 276, 306, 328	665	623, 503, 461, 443, 299, 284	9.98	8.18	6.32	9.06	6.40	7.98	6.35	5.16	0.22	5.37	4.27
Isoscutellarein 7-*O*-[6‴-*O*-acetyl]-allosyl	39.1	230, 276,	693	651, 633, 609, 489,	24.18	4.56	0.81	1.78	nd	4.73	0.85	1.09	0.31	nd	nd

(1→2)-[6″-*O*-acetyl]-glycoside		308, 328		471, 429, 285											
4′-*O*-Methylisoscutellarein 7-*O*-[6‴-*O*-acetyl]-allosyl(1→2)glucoside	40.5	230, 280, 306, 330	707	665, 647, 605, 545, 503, 299, 284	4.81	nd	nd	nd	nd	0.98	nd	0.91	nd	nd	nd
**Hypolaetin derivatives**															
3′-*O*-Methylhypolaetin 7-*O*-allosyl(1→2)glucoside	18.8	230, 256, 276, 300, 342	639	477, 315	nd	1.42	nd	nd	nd	nd	nd	nd	nd	nd	nd
Hypolaetin 7-*O*-[6‴-*O*-acetyl]-allosyl(1→2)glucoside	19.6	230, 254, 276, 300, 344	667	625, 607, 505, 463, 445, 301	1.22	nd	nd	nd	nd	nd	0.41	0.38	nd	nd	nd
3′-*O*-Methylhypolaetin 7-*O*-[6‴-*O*-acetyl]-allosyl(1→2)glucoside	24.5	230, 256, 276, 300, 342	681	639, 621, 519, 501, 459, 441, 315, 301	3.11	3.16	1.89	3.18	2.12	3.06	1.67	2.57	nd	1.85	1.52
4′-*O*-Methylhypolaetin 7-*O*-[6‴-*O*-acetyl]-allosyl(1→2)-[6″-*O*-acetyl]-glucoside	40.4	228, 256, 276, 302, 340	723	681, 663, 639, 621, 477, 315, 301	nd	1.74	1.66	1.95	nd	1.26	0.56	0.92	nd	nd	nd

nd – not detected

Table S3 (a) Eigen values from PCA analysis

Axis	Eigen value	% explained	Histogram	% cumulated
1	3.500528	43.76%		43.76%
2	2.120761	26.51%		70.27%
3	0.837948	10.47%		80.74%
4	0.717971	8.97%		89.72%
5	0.489508	6.12%		95.83%
6	0.238772	2.98%		98.82%
7	0.092445	1.16%		99.97%
8	0.002067	0.03%		100.00%
Tot.	8.000000	-	-	-

Factor Loadings [Communality Estimates]

Attribute	Axis_1		Axis_2		Axis_3		Axis_4		Axis_5		Axis_6		Axis_7		Axis_8	
-	Corr.	% (Tot. %)	Corr.	% (Tot. %)	Corr.	% (Tot. %)	Corr.	% (Tot. %)	Corr.	% (Tot. %)	Corr.	% (Tot. %)	Corr.	% (Tot. %)	Corr.	% (Tot. %)
5-Caffeoylquinic acid	0.1397	*2 % (2 %)*	-0.9003	*81 % (83 %)*	0.1201	*1 % (84 %)*	-0.0519	*0 % (85 %)*	0.3163	*10 % (95 %)*	0.1852	*3 % (98 %)*	-0.1353	*2 % (100 %)*	-0.0097	*0 % (100 %)*
Phenylethanoides	-0.5173	*27 % (27 %)*	0.0747	*1 % (27 %)*	0.8113	*66 % (93 %)*	0.2446	*6 % (99 %)*	-0.0392	*0 % (99 %)*	0.0745	*1 % (100 %)*	0.0416	*0 % (100 %)*	0.0038	*0 % (100 %)*
Luteolin derivatives	0.7611	*58 % (58 %)*	0.1841	*3 % (61 %)*	0.2169	*5 % (66 %)*	-0.5458	*30 % (96 %)*	-0.0540	*0 % (96 %)*	0.1573	*2 % (99 %)*	0.1180	*1 % (100 %)*	-0.0134	*0 % (100 %)*
Apigenin derivatives	0.6609	*44 % (44 %)*	-0.4403	*19 % (63 %)*	-0.0705	*0 % (64 %)*	0.2618	*7 % (70 %)*	-0.5152	*27 % (97 %)*	0.1726	*3 % (100 %)*	-0.0240	*0 % (100 %)*	0.0043	*0 % (100 %)*
Chrysoeriol derivatives	0.7465	*56 % (56 %)*	0.5742	*33 % (89 %)*	0.2091	*4 % (93 %)*	0.1267	*2 % (95 %)*	-0.0539	*0 % (95 %)*	-0.1477	*2 % (97 %)*	-0.1675	*3 % (100 %)*	-0.0208	*0 % (100 %)*
Isoscutellarein derivatives	0.9604	*92 % (92 %)*	0.0185	*0 % (92 %)*	0.1820	*3 % (96 %)*	-0.1249	*2 % (97 %)*	0.1382	*2 % (99 %)*	-0.0779	*1 % (100 %)*	-0.0468	*0 % (100 %)*	0.0348	*0 % (100 %)*
Hypolaetin derivatives	0.5380	*29 % (29 %)*	-0.7551	*57 % (86 %)*	0.1063	*1 % (87 %)*	0.1613	*3 % (90 %)*	0.0409	*0 % (90 %)*	-0.2886	*8 % (98 %)*	0.1343	*2 % (100 %)*	-0.0103	*0 % (100 %)*
Other derivatives	0.6544	*43 % (43 %)*	0.4202	*18 % (60 %)*	-0.1585	*3 % (63 %)*	0.4810	*23 % (86 %)*	0.3096	*10 % (96 %)*	0.1822	*3 % (99 %)*	0.0981	*1 % (100 %)*	-0.0026	*0 % (100 %)*
Var. Expl.	3.5005	*44 % (44 %)*	2.1208	*27 % (70 %)*	0.8379	*10 % (81 %)*	0.7180	*9 % (90 %)*	0.4895	*6 % (96 %)*	0.2388	*3 % (99 %)*	0.0924	*1 % (100 %)*	0.0021	*0 % (100 %)*

Eigen vectors -- Factor Scores

Attribute	Mean	Std-dev	Axis_1	Axis_2	Axis_3	Axis_4	Axis_5	Axis_6	Axis_7	Axis_8

**5-Caffeoylquinic acid**	4.257811	1.928468	0.074648	-0.618250	0.131205	-0.061262	0.452097	0.379028	-0.444945	-0.212597
**Phenylethanoides**	54.166640	17.990714	-0.276463	0.051288	0.886299	0.288627	-0.055986	0.152400	0.136968	0.083579
**Luteolin derivatives**	2.080664	2.484197	0.406807	0.126451	0.236986	-0.644192	-0.077177	0.322002	0.388054	-0.295204
**Apigenin derivatives**	0.987191	0.792058	0.353237	-0.302320	-0.077010	0.308994	-0.736436	0.353230	-0.078849	0.095350
**Chrysoeriol derivatives**	0.268562	0.406152	0.398993	0.394310	0.228432	0.149494	-0.076969	-0.302310	-0.551033	-0.458085
**Isoscutellarein derivatives**	29.371897	26.509148	0.513318	0.012688	0.198797	-0.147354	0.197556	-0.159498	-0.153809	0.766149
**Hypolaetin derivatives**	3.241232	1.729082	0.287555	-0.518495	0.116150	0.190319	0.058528	-0.590625	0.441548	-0.227014
**Other derivatives**	0.623293	0.523802	0.349784	0.288566	-0.173130	0.567625	0.442495	0.372810	0.322803	-0.056722

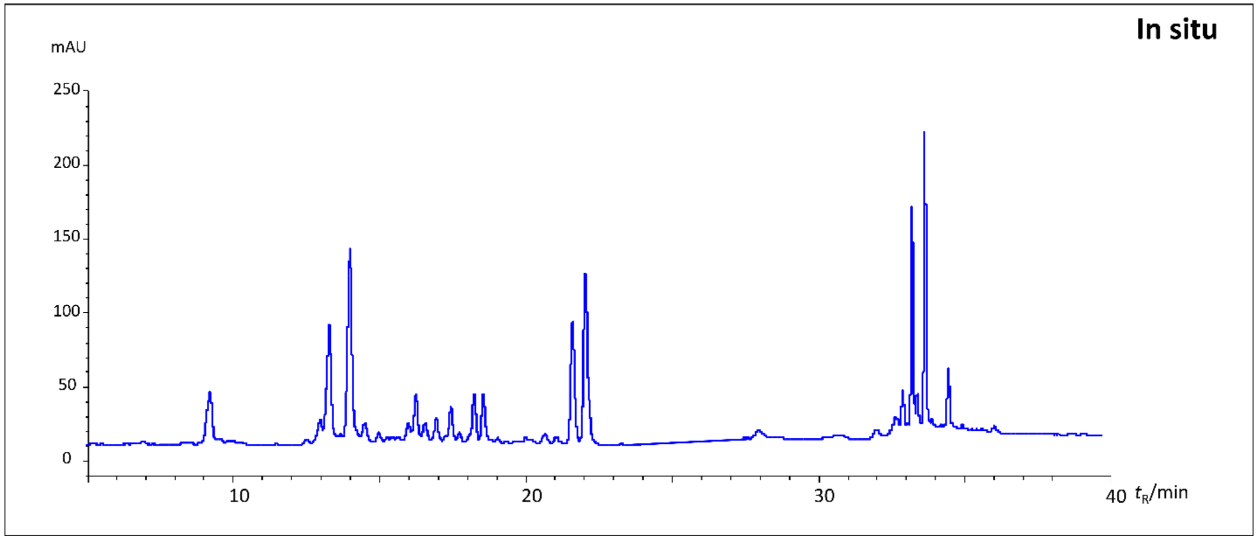
In situ
mAU
250
200
150
100
50
0
10
20
30
40
t_R/min

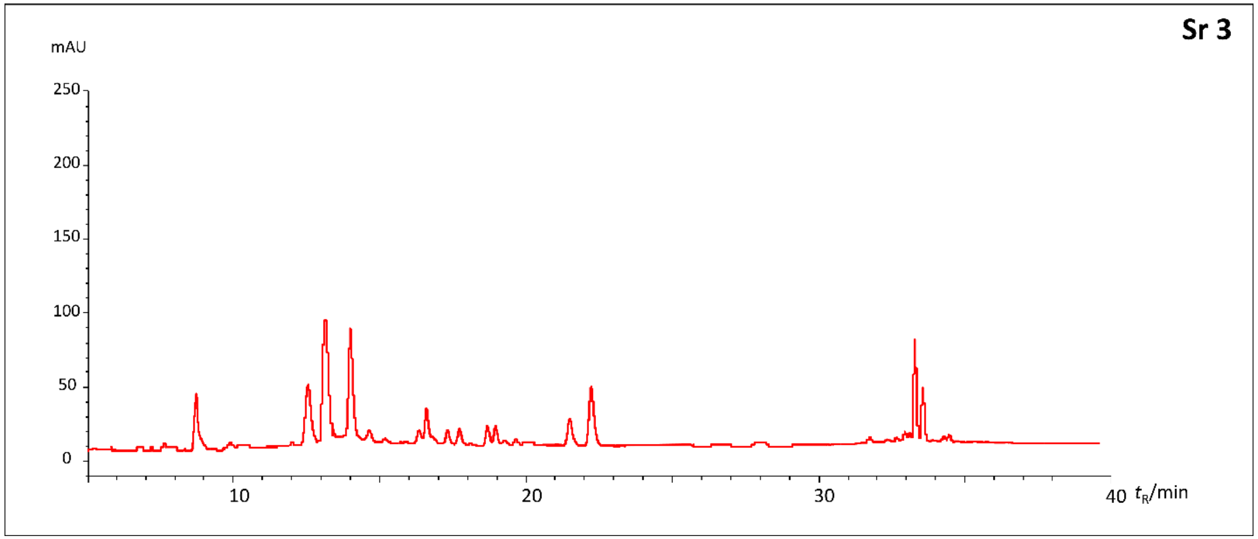
Sr 3
mAU
250
200
150
100
50
0
10
20
30
40
t_R/min

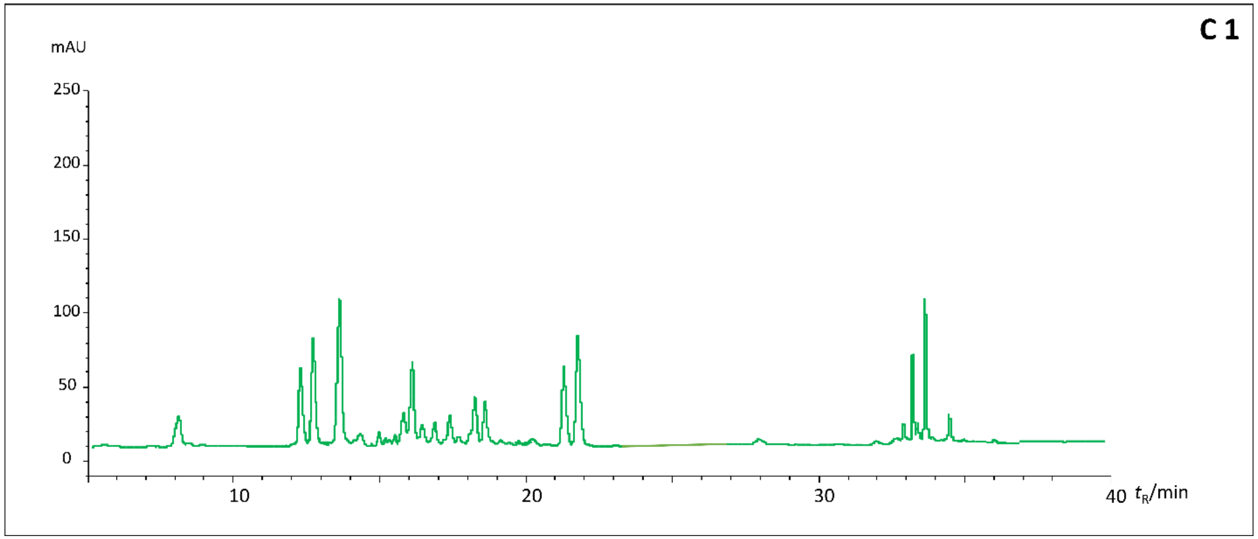

Figure S1. HPLC-DAD chromatograms at 330 nm for in situ sample, PGR supplementation (Sr_3) and AC supplementation (C_1)
